# Anti-Typhoid Vaccination

**Published:** 1909-09-18

**Authors:** 


### MEDICINE.

### ANTI-TYPHOID VACCINATION.

THE question of the value of vaccine inoculations against typhoid fever and the analogous question of the treatment of patients suffering from typhoid fever with vaccine have been worked at by the officials of the Royal Army Medical Corps more fully, probably, than by any other body of men. Each year brings advances in the technique, one of the numerous papers on the subject being that by Major H. W. Grattan and Captain A. L. Webb, read before the United Services Medical Society and printed *in extenso* in the *Journal of the Royal Army Medical Corps*. Although medical practitioners do not actually need a detailed acquaintance with bacteriological methods, nevertheless it enables them to follow the *rationale* of vaccine treatment when they have a clear idea of the process by which a vaccine is prepared. The following is a summary of the various steps of the technique required in making an anti-typhoid vaccine, which consists of a sterilised broth culture of the typhoid bacillus. The broth is ordinary nutrient broth brought to a reaction of +10 (Eyre's scale)—that is to say, it is made alkaline to a certain definite degree. It is sterilised in the autoclave for a minimum period of three-quarters of an hour at 120° C. After cooling over night, it is incubated for 48 hours at 37° C., and then kept for two or three days at room temperature in the dark to prove its sterility. The broth is not sterilised in bulk, but in flasks containing not more than 300 c.c.

The purity of the culture is verified by plating, by titration with an anti-typhoid serum, by direct microscopical examination, and by cultural tests. The flasks are then inoculated and incubated for 48 hours at 37° C., and are placed in a horizontal position in the incubator. A dense growth of typhoid bacilli is obtained with the flasks in this position, owing to the fact that the typhoid bacillus grows more readily in the presence of an abundant supply of oxygen. All the apparatus, with the exception of the bottles, is sterilised in the autoclave for three-quarters of an hour at 120° C. The bottles are sterilised by dry heat.

The contents of a series of flasks are then mixed and measured in a graduated jar; a batch of culture as a rule consists of about 4 litres before it is diluted. Measured volumes of culture are then run off into suitable flasks by means of "two-way tubes." These tubes are then removed, and samples of the growth taken and tested for purity. This precaution is necessary in the preparation of all vaccines, but more especially so when a large quantity is prepared from many flasks. The flasks are closed with their original plugs, which are then covered with waxed paper. This is perforated in order that an even pressure may be maintained, thus ensuring that the temperature of the culture will correspond with the control flask in the bath. The flasks are then placed in a water-bath at a temperature of 45° C. A thermometer placed in the control flask will enable one to ascertain the exact temperature of the culture.

The bath is kept at a temperature of 45° C. for half an hour to allow the temperature of the culture to rise to that of the bath. The temperature of the whole is then gradually raised until the thermometer inside the control flask stands at 53° C. After an hour and ten minutes at 53° C., during which time the flasks are repeatedly shaken, they are taken out of the bath and cooled down. The reason for this cooling is that it has been found by experiment that the potency of the culture is to a large extent destroyed if the antiseptic is added when the culture is hot.

The strength of the culture is then estimated by the "counting method." The technique is as follows: A small pipette is taken and a mark made at a convenient distance from the end; blood is then drawn up to the mark, and this volume of blood is washed out into citrate solution in a small glass capsule. After centrifuging, the supernatant clear fluid is pipetted off, the remaining cells are then twice washed with normal saline, and diluted with a small quantity of this fluid. The culture is then taken up to the same mark in the pipette used for taking the blood, and this is well mixed with the washed cells and saline. Wet film preparations are then made by placing small drops of the preparation on clean sides, dropping cover-glasses on, and ringing them with vaseline.

A series of successive "fields" are passed in view by means of a mechanical stage, and in order to avoid eye-strain the field is narrowed by means of two glass filaments fixed on a disc which is dropped into the eye-piece of the microscope. The number of bacilli and red cells are noted in each field, and not less than 100 fields, or 1,000 red cells, are counted. The calculation is based on the fact that there are 5,000,000,000 red cells in 1 c.c of blood, and by rule of three we can estimate the number of bacilli in 1 c.c. of culture, because we are comparing an equal quantity of a culture of unknown strength with an equal quantity of blood of a known strength. For example, if the count gave 1,500 red cells and 300 bacilli, then the strength of the vaccine would be 1,000,000,000 per c.c. After the culture has cooled down, samples are again taken to see if the bacilli have been killed by the heating. The preservative, which is 0.25 per cent. pure lysol, is now added in the form of a 10-per-cent. solution in distilled water. Two-way tubes are then fitted to the flasks, and the rubber corks and tops of the flasks sealed with wax.

As it has been thought advisable to have a vaccine of a standard strength, and at the same time have the dose in a convenient form, the strength has been fixed at 1,000,000,000 per c.c.; so in cultures stronger than this, diluting fluid is added in sufficient quantity to reduce it to standard strength. The diluting fluid consists of sterile broth + 0.25 per cent. lysol. The following day the diluted vaccine is tested for sterility. After a minimum period of five days, the tests proving sterile, the vaccine is bottled. The technique of bottling is as follows:

The glass guard and pipette are connected to one limb of the two-way tube by means of rubber and glass connections; by the aid of a hand-blower the operator can maintain a constant pressure in the flask, and a suitable flow of vaccine through the pipette. As each bottle is filled it is sealed.

On a subsequent date certain bottles are tested for sterility; two broth tubes and an agar slope are inoculated from each bottle, and ærobic and anærobic cultures made. After a minimum period of five days' incubation, the tests proving sterile, the vaccine is passed as fit for issue. It is labelled with its serial number, the date on which its sterility was verified, the dose in minims and c.c., and the date after which it must not be used, *i.e.* three months from the date of manufacture. It has been found by experiment that the vaccine deteriorates with age. Copies of instructions containing an account of how to deal with the vaccine and what symptoms may be expected after its use are sent with each batch. The dose given is 500,000,000, followed after an interval of ten days by a second dose of 1,000,000,000. The vaccine is given by subcutaneous injection; a convenient spot for the inoculation is the outer surface of the arm on a level with the insertion of the deltoid.

The clinical symptoms which result from anti-typhoid inoculations are subject to considerable individual variations. They may be divided into constitutional and local.

1. *Constitutional Symptoms.*—Some degree of malaise may be expected in every case. Occasionally only there may be a tendency to faintness and perhaps a definite rigor. These symptoms may be expected between the first and sixth hour. When they are at all severe it is the rule for them to come on before the expiration of the third hour. These preliminary symptoms are followed by a certain amount of fever. The average temperature which is attained is about 101° F.; in exceptional cases the temperature may rise as high as 103° F. The fever generally passes off completely at the end of eighteen to twenty-four hours, but in exceptional cases it may persist for about another twenty-four hours.

2. *Local Symptoms.*—In every case a certain amount of local tenderness will develop. This will generally begin to make itself felt about five to six hours after the inoculation. Somewhat later a red blush will appear around the site of inoculation. The local tenderness will be at its worst in about eighteen hours. In many cases the skin will then be red over an area of four to five inches square, and lines of injected lymphatics will sometimes be traceable radiating from the site of inoculation. There may, in addition, be slight tenderness in the armpit.

The most recent statistics published on the subject are to be found in the February number of the *Journal of the Royal Army Medical Corps*, in a paper by Brevet Lieut.-Col. W. B. Leishman.

The total strength of the sixteen units under observation was 12,083. Of these 5,473 were inoculated, amongst whom twenty-one cases of enteric occurred with two deaths. The remaining 6,610 non-inoculated men served as a control; amongst these occurred no fewer than 187 cases of enteric with twenty-six deaths.

The case incidence per 1,000 amongst the "exposed" units, *i.e.* in which cases of enteric had occurred, was 6.6 amongst the inoculated and 39.5 amongst the non-inoculated.

Still more striking results are shown by omitting the Royal Fusiliers (the unit inoculated with the old vaccine), the case incidence per 1,000 being 3.7 amongst the inoculated and 32.8 amongst the non-inoculated. The old vaccine was heated to 62° C., which was found to have a deleterious effect on its immunising properties.

The comparison of these results with former statistics on the protective effect of the old vaccine seems to confirm amply the experimental findings, that an over-heated vaccine, a vaccine to which an antiseptic has been added when hot, or a vaccine which has been kept for more than three months, is very much reduced in efficacy.

